# Amputation-Free Survival, WIfI Stage, and GLASS Classifications in Distal Crural or Pedal Bypass for Chronic Limb-Threatening Ischemia

**DOI:** 10.3390/jcm13226649

**Published:** 2024-11-06

**Authors:** Corinne Kohler, Kristina Gaizauskaite, Konstantinos Kotopoulos, Drosos Kotelis, Jürg Schmidli, Vladimir Makaloski, Salome Weiss

**Affiliations:** Department of Vascular Surgery, Inselspital, Bern University Hospital, University of Bern, Freiburgstrasse, 3010 Bern, Switzerland; corinne.kohler@insel.ch (C.K.); kristina.gaizauskaite@spital.so.ch (K.G.); konstantinos.kotopoulos@insel.ch (K.K.); drosos.kotelis@insel.ch (D.K.); juerg.schmidli@extern.insel.ch (J.S.); salome.weiss@insel.ch (S.W.)

**Keywords:** distal bypass, pedal bypass, chronic limb-threatening ischemia (CLTI), peripheral artery disease (PAD), revascularization

## Abstract

**Background**: Chronic limb-threatening ischemia (CLTI) is a severe condition with high risks of amputation and mortality, especially in patients with distal crural or pedal artery disease. Despite advances in endovascular techniques, bypass surgery remains crucial for patients with CLTI. This study aimed to investigate amputation-free survival, Wound, Ischemia, and foot Infection (WIfI) staging, and Global Limb Anatomic Staging System (GLASS) classifications in patients undergoing distal crural or pedal bypass for CLTI. **Methods**: This retrospective study analyzed all patients who underwent distal crural or pedal bypass for CLTI in a tertiary vascular centre from January 2010 to December 2019. The data were collected from hospital records and preoperative imaging. WIfI stages and GLASS classifications were determined for each patient, and the primary endpoint was amputation-free survival. Secondary outcomes included bypass patency, 30-day morbidity, and mortality. **Results**: We identified 31 bypasses performed on 29 patients with a median age of 67 years (79% male). Preoperatively, 94% of limbs were staged GLASS III and 55% were classified WIfI stage 4. Failed endovascular revascularization preceded bypass surgery in 65% of the cases. Thirty-day mortality was 3% (n = 1) and 30-day major amputation rate was 10%. Primary patency was 87%, and secondary patency was 94% at 30 days. Median duration of follow-up for survival was 59 months with a mean follow-up index (FUI) of 0.99 ± 0.05, and for major amputation and bypass patency 54 months (mean FUI 0.9 ± 0.19 and 0.85 ± 0.28, respectively). At one year, amputation-free survival was 58%, decreasing to 45% at two years, 39% at three years, and 32% at five years. Most major amputations occurred in WIfI stage 4 patients, but 53% of WIfI stage 4 and 80% of WIfI stage 3 patients were alive without major amputation after one year. **Conclusions**: Distal crural and pedal bypasses are essential for limb salvage in high-risk CLTI patients, particularly those with failed prior revascularization. However, the procedure is associated with limited long-term amputation-free survival. WIfI and GLASS classifications are useful for stratifying risk and guiding treatment, but outcomes suggest the need for individualized care strategies. Further research into perioperative management and alternative interventions is warranted to improve long-term outcomes in this population.

## 1. Introduction

Chronic limb-threatening ischemia (CLTI) is a challenging condition associated with a high risk of limb loss and death. Among CLTI patients, the number of those with distal disease including the crural and pedal arteries has grown over the past decades, mainly due to the increased prevalence of diabetes [1]. However, because of fragile and severely calcified small-diameter arteries and poor runoff vessels, the treatment of distal disease has remained a challenge for both endovascular specialists and bypass surgeons. Despite considerable advances in endovascular techniques and the recently published randomized BEST-CLI and BASIL-2 trials [2,3] comparing outcomes of endovascular and bypass revascularisation, the role of bypass surgery for very distal arterial disease continues to be a matter of debate.

The one-year risk of major amputation in CLTI can be estimated using the Wound, Ischemia, and foot Infection (WIfI) classification [4,5]. The WIfI classification classifies wound, ischemia, and foot infection, and the grades from each category result in a clinical WIfI stage ranging from 1 (very low risk of amputation) to 4 (high risk of amputation) [4]. For classifying the anatomical pattern of CLTI, the global limb anatomic staging system (GLASS) has been recommended [5]. GLASS is based on the concept of a target arterial path (TAP), defined as the optimal in-line pathway from the groin to the foot. The separately graded femoropopliteal (FP) and infrapopliteal (IP) segments of the TAP result in a GLASS stage from I to III—reflecting low, intermediate, and high anatomic complexity. The success of endovascular revascularization correlates with the GLASS classification [5]. However, this has not been shown for bypass surgery [6]. Further, the inframalleolar/pedal descriptor of the GLASS classification (P0-P2) describes outflow disease, but is currently not considered in the primary assignment of the GLASS stage, and it is yet unknown how it should be incorporated. While the current guidelines recommend bypass surgery over endovascular revascularization in average risk patients with high complexity disease (GLASS III) as well as those with a high risk of amputation (WIfI stages 3 and 4) [5], data on WIfI and GLASS classification in patients undergoing very distal bypass and their correlation to outcomes are scarce. The aim of this study was to analyze amputation-free survival, WIfI stage, and GLASS classification in patients who underwent distal crural or pedal bypass for CLTI.

## 2. Material and Methods

In this retrospective single-centre series, consecutive patients who underwent distal crural and pedal bypass from January 2010 to December 2019 at a tertiary referral centre were analyzed. All patients had already given written informed consent for the further use of their health-related data, as requested by the local ethics committee, who approved this study (decision number 2020-01422). Data were collected from hospital records and available imaging studies. The Vascular Quality Initiative (VQI) Cardiac Risk Index was calculated using the VQI risk calculator (https://svs-vqi.shinyapps.io/CRICalculators/). Follow-up data were gathered until the end of June 2024, and completeness of follow-up was assessed using the follow-up index [7].

### 2.1. Definitions

CLTI was diagnosed according to current guidelines [5]. Distal crural bypass was defined as a bypass with a distal anastomosis in the distal third of the posterior or anterior tibial or peroneal artery. Pedal bypass was defined as a bypass with a distal anastomosis to the dorsal pedal artery, the common plantar artery, or the medial or lateral plantar artery.

Primary patency was defined as an intervention-free patent bypass observed in duplex ultrasound. Primary-assisted patency was defined as a patent bypass after a surgical or endovascular intervention to prevent occlusion. Secondary patency was defined as patent bypass after intervening surgically or by endovascular means due to bypass occlusion.

### 2.2. WIfI and GLASS Classification

All limbs were retrospectively staged by two authors (KG and CK) using the Society for Vascular Surgery WIfI classification [4]. For wound grading (W), preoperative photo documentation was used, which is meticulously performed for all wounds in vascular patients at our centre. For ischemia grading (I), preoperative ankle-brachial indices, ankle systolic pressures, toe pressures, and transcutaneous oxygen pressures were checked. For grading foot infection (fI), wound photo documentation, vital parameters, laboratory values, and physicians’ reports were considered. Based on W, I, and fI grades, the WIfI stage was determined for every limb.

All preoperative digital subtraction angiography images were retrospectively reviewed, and the GLASS class was determined according to the target arterial path (TAP), using the femoropopliteal and infrapopliteal disease grades. The GLASS inframalleolar/pedal descriptor was documented, although not considered in the primary GLASS classification [5]. The latter included stages P0 (target artery crossing into the foot, with intact pedal arch), P1 (target artery crossing into the foot, with absent or severely diseased pedal arch), and P2 (no target artery crossing the ankle into the foot).

### 2.3. Endpoints

The primary endpoint was amputation-free survival. Secondary endpoints were the 30-day amputation rate, 30-day mortality and morbidity including myocardial infarction, stroke, and perioperative complications (groin lymphatic complications, wound infection and bleeding requiring intervention), reintervention, bypass patency, and long-term mortality.

### 2.4. Surgical Treatment

The decision to perform distal crural or pedal bypass was made by an interdisciplinary vascular team, often after the failure of endovascular revascularization. The site of the proximal and distal anastomoses was chosen at the discretion of the treating vascular surgeon based on previous imaging and graft availability. Whenever available, the great saphenous vein (GSV) was the first choice; otherwise, arm veins were harvested. If no suitable vein graft material was available, a polytetrafluorethylen (PTFE) graft (Bard Peripheral Vascular Inc., Tempe, AZ, USA) was used. If possible, the GSV was used in situ with retrograde in situ valvulotomy and ultrasound-guided side branch ligation. For ex situ vein grafts, angioscopically directed valvulotomy was usually performed, and the vein was used in a non-reversed fashion, minimizing calibre mismatch. In case a PTFE graft was used, the distal anastomotic area was enlarged with a Linton patch plasty (venous patch plasty of the target artery, followed by a longitudinal incision of the proximal two-thirds of the patch, in which the distal bypass anastomosis is performed). Heparin (100 IU/kg body weight) was administered intravenously before clamping. Intraoperative completion angiography was performed, and if identified, technical faults were corrected.

### 2.5. Postoperative Treatment and Surveillance

Postoperatively, all patients received a combination of one antiplatelet agent (aspirin or clopidogrel) and heparin, which was switched to an oral anticoagulant (phenprocoumon or direct oral anticoagulant) in a therapeutic dose before discharge. Antiplatelet therapy was usually continued for three months in addition to oral anticoagulation. Perioperative intravenous iloprost was applied at the discretion of the surgeon. Standard follow-up visits including clinical examination and duplex ultrasound to assess bypass patency were performed at 4 weeks, and at three, six, and twelve months, and usually annually thereafter. Open surgical or endovascular reinterventions were performed as deemed necessary by the treating vascular team to maintain or restore bypass patency.

### 2.6. Statistical Analysis

Data are presented as median values and (interquartile) range, or where appropriate as mean values ± standard deviation for continuous variables and as absolute numbers and percentages for categorical variables. The time-to-event data were analyzed using the Kaplan–Meier technique. Statistical analyses were performed using SPSS Statistics 22.0.0.0 (IBM, Armonk, NY, USA) and R-Studio (RStudio Inc., Boston, MA, USA).

The manuscript was prepared according to the Strengthening the Reporting of Observational Studies in Epidemiology (STROBE) checklist [8].

## 3. Results

From 2010 to 2019, 41 distal crural and pedal bypasses were performed in 39 patients. Nine of these bypass cases were excluded because they were performed in patients with acute limb ischemia, and one because the bypass was needed due to traumatic crural vessel lesion. Thirty-one bypasses in 29 patients were performed for CLTI and included in this study. Patients had a median age of 67 years (range 21–85) and 79% were male. In 65% of limbs (20/31), previous endovascular revascularization techniques had failed (including antegrade and retrograde wire crossing of chronic total occlusions of the crural arteries). In 16% of limbs (5/31), previous more proximal bypass surgery had failed. Further baseline characteristics are presented in Table 1 for all patients as well as stratified for postoperative major amputation.

Limbs were retrospectively classified as WIfI stage 4 in 55%, stage 3 in 16%, and stage 2 in 29%. Overall, 77% of patients were treated due to tissue loss and the remaining due to ischemic rest pain (Table 1). The majority of limbs were GLASS stage III (94%) and 90% had either severely diseased or absent pedal arch or no target artery crossing the ankle into the foot at all (GLASS inframalleolar/pedal modifier P1 or P2; Table 2).

A total of 16 bypasses were performed to the distal third of the crural arteries, and 15 were pedal bypasses. The GSV was the most frequently used graft material (21/31, 68%). Prosthetic graft material was used in four patients (Table 3).

### 3.1. Thirty-Day Outcomes

One patient (3%) died within 30 days after surgery with a patent bypass and without major amputation. This patient had undergone coronary artery bypass nine days prior to pedal bypass and suffered from pericardial tamponade on day 15 after pedal bypass surgery with consecutive resuscitation and hypoxic brain damage leading to death on day 16.

Three major amputations (10%) were performed after 2, 14, and 25 days. Two had a patent GSV bypass, but amputation became necessary due to severe tissue damage (both initially staged WIfI 4). One suffered from PTFE bypass occlusion on the first postoperative day with consecutive surgical bypass revision and re-occlusion one day later. The latter patient was initially staged WIfI 2 (initially no wound, no infection, but ischemia grade 3). All three patients undergoing early major amputation had been classified GLASS III.

Fourteen minor amputations were performed within 30 days. Overall, four bypasses occluded within 30 days, but only the aforementioned one resulted in major amputation within 30 days. Of the remaining three early bypass occlusions, one patient with an arm vein bypass underwent surgical thrombectomy with patch plasty of the distal anastomosis to the plantar artery on postoperative day 1 with no consecutive major amputation; one patient with a PTFE bypass underwent surgical thrombectomy with local lytic therapy but suffered from re-occlusion 45 days later resulting in major amputation; and in one patient with an early GSV bypass occlusion the bypass was abandoned and endovascular deep venous arterialisation was tried, resulting in major amputation after 3.6 months. Twenty-seven bypasses remained open without any intervention in 30 days (30-day primary patency 27/31, 87%; secondary patency 29/31, 94%).

No perioperative myocardial infarction or stroke was observed. Two patients experienced lymphatic complications in the groin, and one had a wound infection. No postoperative bleeding requiring intervention occurred.

### 3.2. Long-Term Outcomes

Median follow-up for survival was 59 months (interquartile range [IQR] 15–87) with a mean follow-up index (FUI) of 0.99 (±0.05). For major amputation, follow-up data were available for a median of 54 months (IQR 11–91) with a mean FUI of 0.9 (±0.19). For bypass patency, the median follow-up was 54 months (IQR 10–82) with a mean FUI of 0.85 (±0.28).

**Figure 1:**
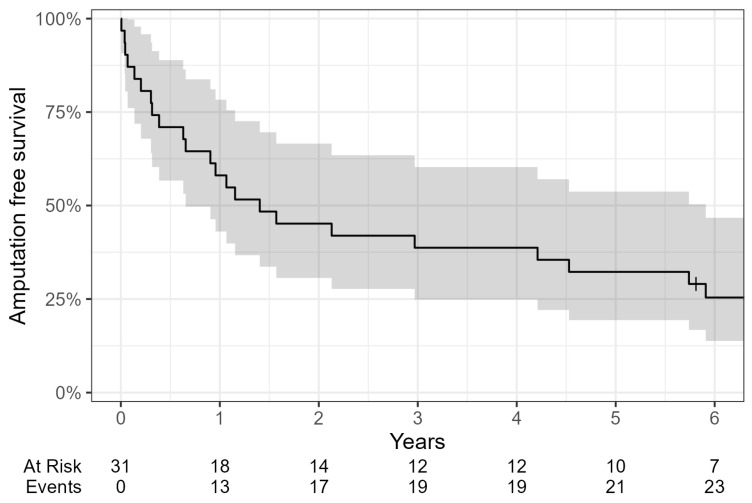
Amputation-free survival (major amputations).

**Figure 2:**
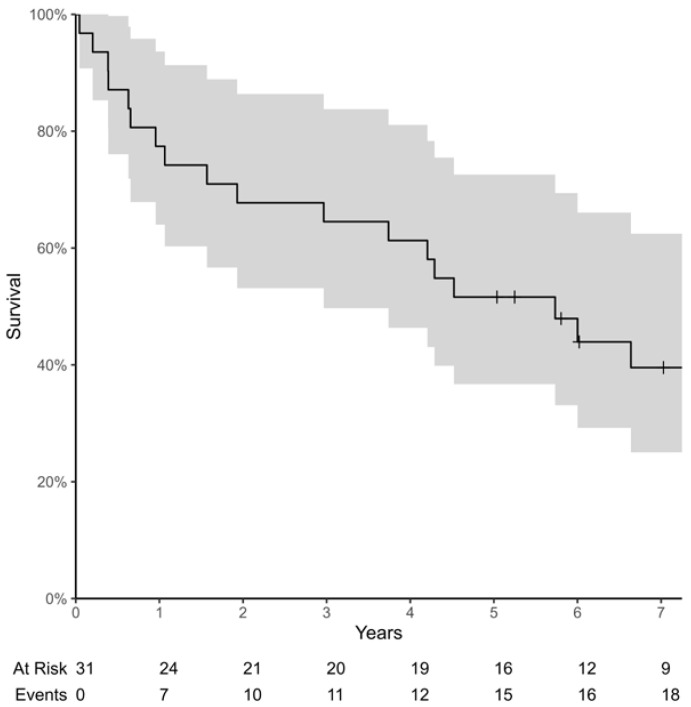
Survival.

During follow-up, nine additional major amputations were performed after a median of 13 months (range 1.6–94) after bypass surgery of the respective limb. Four of these major amputations occurred within one year of bypass surgery (plus three within 30 days) and five thereafter. Twenty-one patients died after a median of 45 months (range 2–160). The estimated major amputation-free survival at 1, 2, 3, and 5 years was 58%, 45%, 39% and 32% (Figure 1). The estimated survival at 1, 2, 3, and 5 years was 77%, 68%, 65%, and 52%, respectively (Figure 2). Bypass patency rates are presented in Table 4.

### 3.3. WIfI and GLASS

**Figure 3:**
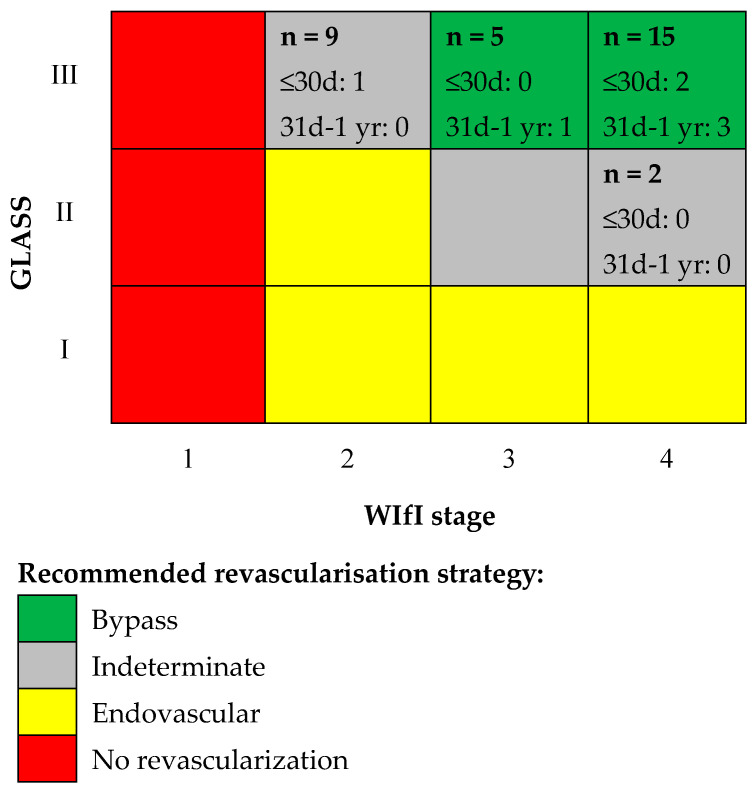
Adapted from the Global Vascular Guidelines on the Management of Chronic Limb-Threatening Ischemia. In each box, the total number of limbs per group and the number of major amputations per group within 30 days and one year are presented; the legend on the right side refers to the preferred revascularisation strategy according to the Global Vascular Guidelines; GLASS = global limb anatomic staging system; WIfI = wound, ischemia, and foot infection.

**Figure 4:**
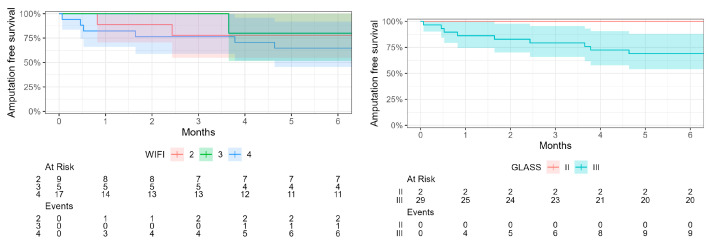
Amputation-free survival stratified by WIfI stages 2–4 (**left** panel) and GLASS classes II and III (**right** panel) during the first six months after bypass surgery.

Figure 3 displays the distribution of cases considering both classifications as well as the number of amputations per group. Based on GLASS and WIfI stages, bypass surgery was the preferred revascularisation strategy for 20 (65%) of patients in our series, according to the Global Vascular Guidelines on the Management of CLTI (assuming average risk). Seven out of overall twelve major amputations were initially WIfI stage 4 (however, five major amputations occurred more than one year after bypass surgery). Amputation-free survival stratified by WIfI and GLASS is depicted in Figure 4. At one year, 9/17 (53%) WIfI stage 4 patients and 8/15 (53%) WIfI 4-GLASS III patients were alive without major amputation, while 4/5 (80%) WIfI stage 3 and 5/9 (56%) WIfI stage 2 patients were alive without major amputation. Looking at major amputations at one year only, the highest rate occurred in WIfI 4 GLASS III patients (5/15, Figure 3).

## 4. Discussion

Distal crural or pedal bypass may be the last option for limb salvage in many patients with CLTI. In our series of 31 distal crural and pedal bypasses for CLTI, we observed a limited major amputation-free survival at 1, 2, 3, and 5 years of 58%, 45%, 39%, and 32%. Almost all limbs had preoperatively been staged GLASS III, and more than 70% were WIfI stage 3 and 4. Most major amputations at one year occurred in WIfI 4 limbs; nevertheless, 53% of WIfI stage 4 patients and 80% of WIfI stage 3 patients were alive without major amputation at one year. Only 56% of WIfI stage 2 patients were alive without major amputation at one year, which was primarily due to a high mortality in this group.

Our indications for bypass surgery were in accordance with the recommendations of the current Global Vascular Guidelines [5]. Based on the WIfI and GLASS stages, 20 limbs fell in the category “open bypass” as the preferred initial revascularisation strategy, and the remaining 11 limbs were categorized into the “indeterminate” group. It has to be considered, however, that distal crural or pedal bypass surgery was not always the initial strategy in our series, but followed endovascular failure in 65% and more proximal bypass failure in 16%. Even more importantly, the Guidelines’ recommendations apply to average-risk patients with suitable autologous vein conduit in the absence of severe pedal disease (GLASS P2). These criteria were not true for all patients included in our series, but often, bypass was the last option. It has especially to be noted that most patients in our cohort may not be considered at average surgical risk, as 76% had a VQI Cardiac Risk Index of >1.8%, which is generally considered average risk for patients undergoing infrainguinal revascularization procedures.

When looking at our primary endpoint of amputation-free survival, the results from our series (58%, 45%, 39%, and 32% at 1, 2, 3, and 5 years) are less favourable as those from comparable study populations. In one of the largest series of pedal bypasses, amputation-free survival was 74%, 60%, 49%, and 41% [9], and in the BASIL-1 trial, it was 72%, 62%, 53%, and 35% [3], at 1, 2, 3, and 5 years, respectively. In comparison, studies including endovascular patients with infrapopliteal disease reported a 2-year amputation-free survival of 86–91% [10] and a 4-year amputation-free survival of 65% [11]. However, endovascular revascularisation had failed in the majority of our patients. Our 30-day mortality rate of 3% is comparable with other series. Specialized single centres have reported 30-day mortality rates as low as 1% in larger cohorts of infrapopliteal [12] or pedal bypass patients [13]. In other reports, including those from the BASIL trials, 30-day mortality was 3.23–7% for infrapopliteal [3,14,15,16] and 5.6% pedal bypass [17].

Several studies have shown that the WIfI stage correlates with the risk of major amputation regardless of revascularisation and revascularisation method [18,19]. This correlation has also been shown for CLTI patients undergoing infrapopliteal bypass specifically [12]. Similarly, in our series, most major amputations occurred in those with WIfI stage 4, but amputation-free survival did not consistently correlate with the preoperative WIfI stage. Although at one year, it was lowest for WIfI 4 limbs (53%), it was higher for WIfI 3 (80%) than for WIfI 2 limbs (56%). Looking at Figure 4, amputation-free survival in the WIfI 2 group falls below the WIfI 3 group after one month. This is due to one early amputation (day 25) in a WIfI 2 patient who suffered two consecutive early PTFE bypass occlusions (no suitable vein conduit) and one early death (day 74). In addition, the low numbers in both the WIfI 2 and 3 groups likely skew the results.

For the correlation of GLASS with limb outcomes, the available evidence is less clear. In a retrospective evaluation of GLASS stages among patients from the BASIL-1 trial, increasing GLASS grade was significantly associated with immediate technical failure and amputation-free survival in the endovascular treatment group but not in the bypass group. Further, the superiority of bypass over endovascular therapy increased as the femoropopliteal GLASS grade increased [6]. However, the inframalleolar descriptor (P0–P2) was not considered, and only a third of all bypasses had an infrapopliteal target [16,20]. Therefore, GLASS grades were more heterogeneous (60% GLASS III and 20% each GLASS I and II) than in our series. In another, more recent series with 211 crural bypasses and 78% GLASS III limbs, no significant difference in any limb-related outcome between GLASS I-II limbs and GLASS III limbs was found [21]. In our series, amputation-free survival during the first six months tended to be lower for GLASS II compared to GLASS III limbs. However, due to the selection of distal crural and pedal bypasses, almost all limbs were GLASS III in our series. As for the inframalleolar descriptor, larger numbers are necessary to draw any conclusions. Poor outflow would be expected to correlate with poor bypass flow. However, while intraoperative bypass flow has been shown to be a predictor of patency, it was not related to limb salvage in crural bypass surgery [22]. Accordingly, in a larger series of crural bypasses, Kobayashi et al. found a significantly lower patency in P2 limbs compared to P0 and P1 but no significant difference in limb salvage between limbs with P0, P1, and P2 inframalleolar modifier [23]. Thus, the role of GLASS in the prediction of limb outcomes after bypass surgery seems yet unclear.

The high mortality in CLTI patients [24] affects amputation-free survival regardless of preoperative WIfI stage and GLASS class. At one and five years, the estimated mortality in our cohort was 23% and 48%, respectively. It has been shown that coronary revascularisation improves 5-year survival in CLTI patients with silent coronary ischemia who underwent lower limb revascularisation [25]. During the study period, we did not test for perioperative silent coronary ischemia, but this may be relevant for improving the survival of these patients in the future.

### Limitations

This study is primarily limited by its small sample size and the retrospective design. The WIfI stages and GLASS grades were retrospectively determined from the available clinical documentation and angiographies. In addition, limbs were not re-staged for WIfI after revascularisation and regularly thereafter. WIfI re-staging may provide further insights in its applicability in bypass surgery, especially since 42% of major amputations in our series occurred beyond one year after the bypass procedure. Further, more patient-centred endpoints such as wound healing and functional outcomes should be looked at in the future. Another limitation is the inclusion of distal crural and pedal bypasses as well as the inclusion of patients without an adequate GSV conduit, which made the series more heterogeneous. According to the Global Vascular Guidelines, patients without available GSV graft should be considered separately [5]. However, in our patients without a GSV conduit, endovascular therapy was not an option or had failed. The inclusion of these patients reflects clinical reality. Further, we did not test for perioperative silent coronary ischemia, which may have given more insights regarding the high mortality in the longer term.

## 5. Conclusions

Patients undergoing distal crural and pedal bypass for CLTI have an inherently high risk of limb loss and death. In our series, amputation-free survival was limited. Most major amputations at one year occurred in WIfI 4 limbs. Nevertheless, more than half of WIfI 4 patients were alive without major amputation at one year. While WIfI and GLASS may aid decision making in these patients, it should remain an individual, patient-centred process.

## Figures and Tables

**Table 1:** Patients with CLTI—characteristics, risk factors, and previous treatment.

N	All 29 Patients (31 Limbs) *	3 Limbs with Major Amputation ≤ 30 Days	9 Limbs with Major Amputation > 30 Days	19 Limbs without Major Amputation
**Demographics**				
Age (years), median [range]	67 [21–85]	77 [69–84]	66 [21–80]	64 [39–85]
Male	23 (79%)	3 (100%)	7 (78%)	15 (79%)
**Preoperative risk factors**				
BMI (kg/m^2^), median [range]	25 [16–34]	24.5 [24–33]	27 [17–34]	25 [16–34]
Hypertension	24 (83%)	3 (100%)	7 (78%)	16 (84%)
Dyslipidemia	20 (69%)	3 (100%)	5 (56%)	13 (68%)
Diabetes mellitus	14 (48%)	1 (33%)	5 (56%)	9 (47%)
Active smoker	10 (34%)	2 (67%)	3 (33%)	6 (32%)
Former smoker	12 (41%)	1 (33%)	2 (22%)	10 (53%)
Cerebrovascular disease	4 (14%)	0 (0%)	1 (11%)	3 (16%)
COPD	3 (10%)	1 (33%)	0 (0%)	2 (11%)
Coronary heart disease	11 (38%)	1 (33%)	3 (33%)	8 (42%)
eGFR (CKD-EPI) < 30 mL/min	5 (17%)	0 (0%)	3 (33%)	2 (11%)
**ASA classification**				
I	0 (0%)	0 (0%)	0 (0%)	0 (0%)
II	5 (17%)	0 (0%)	2 (22%)	3 (16%)
III	15 (52%)	2 (67%)	5 (56%)	8 (42%)
IV	7 (24%)	1 (33%)	2 (22%)	6 (32%)
V	2 (7%)	0 (0%)	0 (0%)	2 (11%)
**VQI Cardiac Risk**				
VQI CRI, median [range]	2.7 [0.3–5.2]	3.1 [1.3–3.7]	2.5 [0.3–4.6]	2.7 [0.4–5.2]
VQI CRI ≤ 1.8%	7 (24%)	1 (33%)	3 (33%)	4 (21%)
VQI CRI > 1.8%	22 (76%)	2 (67%)	6 (67%)	15 (79%)
**Previous medical treatment**				5 (26%)
Aspirin	16 (55%)	3 (100%)	4 (44%)	11 (58%)
Clopidogrel	4 (14%)	0 (0%)	2 (22%)	2 (11%)
Aspirin and Clopidogrel	7 (24%)	0 (0%)	2 (22%)	5 (26%)
Aspirin or Clopidogrel and DOAC	2 (7%)	0 (0%)	1 (11%)	1 (5%)
**Rutherford stage**				
4	7 (23%)	1 (33%)	2 (22%)	4 (21%)
5	21 (68%)	2 (67%)	6 (67%)	13 (68%)
6	3 (10%)	0 (0%)	1 (11%)	2 (11%)
**Previous treatment**				
Failed endovascular revascularisation	20 (65%)	3 (100%)	5 (56%)	11 (58%)
Failed more proximal bypass	5 (16%)	0 (0%)	2 (22%)	3 (16%)

Values are presented as n (%) unless specified otherwise; ASA = American Society of Anesthesiologists; BMI = body mass index; CLTI = chronic limb-threatening ischemia; COPD = chronic obstructive pulmonary disease; CKD-EPI = chronic kidney disease epidemiology collaboration; DOAC = direct oral anticoagulant; eGFR = estimated glomerular filtration rate; VQI CRI = Vascular Quality Initiative Cardiac Risk Index, i.e., the risk of an in-hospital postoperative myocardial infarction, where 1.8% is the average risk of patients undergoing infrainguinal bypass; * number of patients is given here, whereas for Rutherford stage, previous treatment and, in the two columns to the right, the characteristics per number of limbs are given—numbers per limb may add up to more than the total number of patients.

**Table 2:** Preoperative WIfI stage and GLASS classification and resulting major amputations in the respective groups over the entire follow-up period.

Risk of Amputation	WIfI Stage	Number of Limbs (%)	Failed Endovascular Treatment or Failed Proximal Bypass	WIfI “Wound” Grade ≥ 1	WIfI “Ischemia” Grade 3	Major Amputation, n
Very low	Stage 1	0	0	0/0	0/0	0/0
Low	Stage 2	9 (29%)	8/9	2/9	7/9	4/9
Moderate	Stage 3	5 (16%)	3/5	5/5	4/5	1/5
High	Stage 4	17 (55%)	14/17	17/17	16/17	7/17
GLASS classification	II III	2 (6%) 29 (94%)				1/2 11/29
Inframalleolar/pedal descriptor	P0	3 (10%)				
	P1	22 (71%)				
	P2	6 (19%)				

GLASS = global limb anatomic staging system; WIfI = wound, ischemia, and foot infection.

**Table 3:** Surgical details.

		Total = 31 Bypasses
Proximal anastomosis	Common femoral artery	5
	Superficial femoral artery	6
	Deep femoral artery	2
	Popliteal artery	16
	Anterior tibial artery *	1
	Peroneal artery *	1
Distal anastomosis	**Distal third of crural artery**	**16**
	Anterior tibial artery	6
	Peroneal artery	2
	Posterior tibial artery	8
	**Plantar artery**	**4**
	**Dorsal pedal artery**	**11**
Bypass material	**GSV** thereof in situ	**21** 8
	thereof reversed	2
	thereof non-reversed	11
	**Arm vein**	**6**
	**PTFE**	**4**

GSV = great saphenous vein; PTFE = polytetrafluorethylen. * In these two patients, the popliteal artery was not suturable due to heavy calcifications and an already implanted stent, respectively.

**Table 4:** Patency of bypasses (total of n = 31).

	%
**At 30 days**	
Primary patency	87
Primary-assisted patency	87
Secondary patency	94
**At 1 year (Kaplan–Meier estimates)**	
Primary patency	34
Primary-assisted patency	54
Secondary patency	66
**At 3 years (Kaplan–Meier estimates)**	
Primary patency	28
Primary-assisted patency	49
Secondary patency	49

## Data Availability

The original contributions presented in the study are included in the article, further inquiries can be directed to the corresponding author.
